# The HLA Ligandome Comprises a Limited Repertoire of O-GlcNAcylated Antigens Preferentially Associated With HLA-B*07:02

**DOI:** 10.3389/fimmu.2021.796584

**Published:** 2021-12-01

**Authors:** Soumya Mukherjee, Alvaro Sanchez-Bernabeu, Laura C. Demmers, Wei Wu, Albert J. R. Heck

**Affiliations:** ^1^ Biomolecular Mass Spectrometry and Proteomics, Bijvoet Center for Biomolecular Research and Utrecht Institute for Pharmaceutical Sciences, Utrecht University, Utrecht, Netherlands; ^2^ Netherlands Proteomics Centre, Utrecht University, Utrecht, Netherlands

**Keywords:** O-GlcNAcylation modification, HLA, MHC, immunopeptidome, HLA-B*07:02, neo-antigen

## Abstract

Mass-spectrometry based immunopeptidomics has provided unprecedented insights into antigen presentation, not only charting an enormous ligandome of self-antigens, but also cancer neoantigens and peptide antigens harbouring post-translational modifications. Here we concentrate on the latter, focusing on the small subset of HLA Class I peptides (less than 1%) that has been observed to be post-translationally modified (PTM) by a *O*-linked N-acetylglucosamine (GlcNAc). Just like neoantigens these modified antigens may have specific immunomodulatory functions. Here we compiled from literature, and a new dataset originating from the JY B cell lymphoblastoid cell line, a concise albeit comprehensive list of *O*-GlcNAcylated HLA class I peptides. This cumulative list of *O*-GlcNAcylated HLA peptides were derived from normal and cancerous origin, as well as tissue specimen. Remarkably, the overlap in detected *O*-GlcNAcylated HLA peptides as well as their source proteins is strikingly high. Most of the *O*-GlcNAcylated HLA peptides originate from nuclear proteins, notably transcription factors. From this list, we extract that *O*-GlcNAcylated HLA Class I peptides are preferentially presented by the HLA-B*07:02 allele. This allele loads peptides with a Proline residue anchor at position 2, and features a binding groove that can accommodate well the recently proposed consensus sequence for *O*-GlcNAcylation, P(V/A/T/S)g(S/T), essentially explaining why HLA-B*07:02 is a favoured binding allele. The observations drawn from the compiled list, may assist in the prediction of novel *O*-GlcNAcylated HLA antigens, which will be best presented by patients harbouring HLA-B*07:02 or related alleles that use Proline as anchoring residue.

## Introduction

Peptide antigen presentation by the human leukocyte antigen (HLA) to T-cells forms an integral part of the human immune surveillance (1). Characterizing the identity of these HLA peptide antigens is a critical first step to understand their immunomodulatory functions, and may guide important decisions in the development of vaccines used for immunotherapy (2, 3). A major source of therapeutic intervention for cancer patients comes from neoantigens in the HLA Class I ligandome, introduced by cancer induced mutations (4, 5). Mass spectrometry (MS) based immunopeptidomics provides means to identify disease or patient specific HLA antigen presentation, and chart the HLA bound peptide ligandome (6, 7). Next to the peptide sequence and protein of origin, mass spectrometry can also be used to identity post-translation modifications (PTMs) on the antigens. These may constitute another source of “neoantigens”, as several PTMs are recognized as hallmarks of specific diseases, including cancer and autoimmune diseases (8, 9). Peptide antigens carrying such PTMs have been shown to affect or even regulate immune system recognition of the HLA Class I peptides (10–12).

Of all the HLA peptides presented by cells or in a tissue, only a small fraction seems to harbor PTMs (13–18). Several studies have reported on phosphorylated HLA peptides, and even shown that these could be considered Tumor Associated Antigens (TAAs) (19). Since phosphorylation signalling can be drastically dysregulated in cancer, immunity to such modified epitopes can also be lost or gained under disease conditions (19, 20). Modification on serine and threonine residues with β N-acetylglucosamine (*O*-GlcNAc) is known to engage in reciprocal crosstalk with phosphorylation (21), and such a dynamic balance between phosphorylation and *O*-GlcNAcylation is particularly relevant in the nucleus and cytosol, where the enzymes coordinating the addition or removal of *O*-linked N-acetylglucosamine (*O*-GlcNAc) reside. When *O*-GlcNAcylated proteins are degraded *via* the proteasomal route and loaded onto the HLA Class I molecules, the presented peptides can retain the *O*-GlcNAc moiety from the source protein. Notably, aberrant *O*-GlcNAcylation has been shown to correlate with augmented cancer cell proliferation, survival, invasion, and metastasis (22). It has also been reported that HLA peptides can elicit glycopeptide specific T-cell responses (13, 14). Hence, it is important to characterize the HLA ligandome for *O*-GlcNAcylated class I peptides.

HLA class I peptides are generally more difficult to sequence and identify by mass spectrometry, compared to tryptic peptides, as they are relatively small, do not carry a charged C-terminus (R/K in tryptic peptides), and fragment less efficiently into complementary series of *b*- and *y*-ions. Recent advances in immunopeptidomics have substantially improved the identification of HLA peptides, notably the sensitivity and sequence specificity have been improved, making detection of neoantigens more feasible (23, 24). Using hybrid tandem MS fragmentation methods, Marino et al. and Malaker et al. independently identified so far some of the largest sets of *O*-GlcNAc modified HLA Class I peptides (25, 26). Both studies reported a few dozen of unique *O*-GlcNAc modified HLA Class I peptides, but also showed that some of these carried additional glycans, extended by Gal, Gal-NeuAc and even other monosaccharides. Moreover, Malaker et al. reported potent multifunctional T-cell responses to some of these *O*-GlcNAc modified HLA Class I peptides, but not to the unmodified HLA Class I peptide counterpart, and apparently also found more *O*-GlcNAc modified HLA Class I peptides in cancer cells and tissue, compared to non-cancerous cells. To further improve our understanding of the immunological role that *O*-GlcNAc modified HLA Class I peptides might have to play, it is important to examine the properties of these peptides, the source proteins of origin, the HLA loading specificity, and whether these peptides are functionally activating in immune surveillance.

In this report, using hybrid fragmentation MS strategies, we identified a new set of in total 23 *O*-GlcNAc HLA Class I peptides presented by the non-cancerous JY B-lymphoblastoid cell line. We compared this dataset with the earlier reported *O*-GlcNAc HLA Class I peptides described in the literature to collate a total list of 55 *O*-GlcNAcylated HLA Class I peptides. Our compilation includes the glycopeptide sequence, the likely subcellular localisation of the proteins of origin and the predicted HLA allele and their respective binding affinity. We observed substantial congruence between the glycopeptide sequences we report here and the existing datasets of Malaker *et al*, and Marino et al. While comparing these datasets we noticed that *O*-GlcNAc HLA Class I peptides were presented with a marked preference by HLA-B*07:02, which harbors a Pro anchoring site for the P2 position.

## Materials and Methods

### Cell Culture and Isolation of HLA Class I Associated Peptides

The B-lymphoblastoid cell line JY, homozygote in class I alleles (HLA-A*02:01, HLA-B*07:02, HLA-C*07:02) was cultured in RPMI 1640 supplemented with 10% fetal bovine serum, 50 U/mL penicillin, and 50 μg/mL streptomycin. HLA class I peptides were retrieved *via* immunoaffinity purification, as described previously (24, 27). Specifically, HLA class I complexes were immunoprecipitated using the pan-HLA class I mouse monoclonal IgG2a antibody W6/32 (28), and antigen peptides were separated from HLA molecules by elution with 10% (v/v) acetic acid, and filtration over a 10-kDa molecular weight cutoff membrane (Merck Millipore). The HLA class I peptide ligands was freeze-dried, and reconstituted in 0.1% formic acid for further cleanup by C18 STAGE tips (Thermo Fischer Scientific) before LC/MS-MS analysis.

### LC-MS/MS Analysis

The HLA Class I peptides were analyzed using an Ultimate 3000 UHPLC (Thermo Fisher Scientific) coupled to an Orbitrap Fusion Lumos (Thermo Fischer Scientific). The peptides were trapped on (Thermo Fisher Scientific, µ-Precolumn, 300 µm i.d. x 5mm, C18 PepMap100, 5 µm, 100 Å) for 5 min in solvent A (0.1% formic acid in water) before being separated on an analytical column (Agilent Poroshell, EC-C18, 2.7 μm, 50 cm × 75 μm). Solvent B consisted of 80% acetonitrile in 0.1% formic acid. The gradient was as follows: first 5 min of trapping, followed by 100 min gradient from 5% to 40% solvent B. Subsequently, 10 min of washing with 99% solvent B and 10 min re-equilibration with 9% solvent A. The mass spectrometer operated in data-dependent mode. Full scan MS spectra from *m/z* 350 to 1400 were acquired at a resolution of 120,000 in the Orbitrap after accumulation to a target value of 4 × 10^5^ or a maximum injection time of 50 ms.

For global acquisition of the JY immunopeptidome, higher-energy collisional dissociation (HCD) MS/MS spectra were acquired at a resolution of 60,000. Charge states of 2 up to 4 starting at *m/z* 120 were chosen for fragmentation using the MIPS algorithm with 1.2 Da isolation window. The fragmentation was performed using 30% normalized collision energy (NCE) on selected precursors with 30s dynamic exclusion after accumulation of 5×10^4^ ions. Focused characterisation of the *O*-GlcNAcylated immunopeptidome was performed by electron transfer dissociation (ETD) and energy-stepped HCD, triggered when two of the six *O*-GlcNAc signature fragment ions (*m/z* 204, 186, 168, 144, 138, and 126) were detected at > 5% relative abundance, as described previously (29, 30).

### Data Analysis

All RAW files were searched using the PEAKS Studio 10.5 against the Swiss-Prot human database (20,258 entries, downloaded in February 2018) edited with the JY-specific HLA proteins and 20 most abundant FBS contaminants, with no enzyme specificity in the search engine to identify the unmodified HLA ligandome. Precursor ion and MS/MS tolerances were set to 10 ppm and 0.03 Da. Methionine oxidation and cysteinylation were set as variable modifications. Peptides were filtered by precursor tolerance 5 ppm, < 1% FDR, XCorr > 1.7, and peptide rank 1. Only peptides between 8 and 12 amino acid long were selected for further analysis. Glycopeptide assignments were made by searching the same RAW files using Byonic 4.1. Precursor and MS/MS tolerance were set to 10 ppm and 0.03 Da. Peptide loading affinity was predicted with NetMHC 4.1 algorithm for the identified HLA peptide sequences against HLA-A*02:01, HLA-B*07:02 and HLA-C*07:02 allele, without considering potential changes in loading affinity due to *O*-GlcNAcylation. Peptides were considered binders when % Rank < 2. We classified the binding predictions for the *O*-GlcNAc HLA peptides as strong (IC50 ≤ 50 nM), regular (50 nM < IC50 ≤ 500 nM) and weak (500 nM < IC50 ≤ 5000 nM) binders for peptide sequence. Gene ontology (GO) analysis of the source proteins of the *O*-GlcNAcylated HLA class I peptides was performed by functional annotation tool DAVID (31). The mass spectrometry proteomics data have been deposited to the ProteomeXchange Consortium *via* the PRIDE (32) partner repository with the dataset identifier PXD028874.

## Results and Discussion

### Triggered MS Detection of O-GlcNAcylated HLA Peptide Ligands

**Figure 1:**
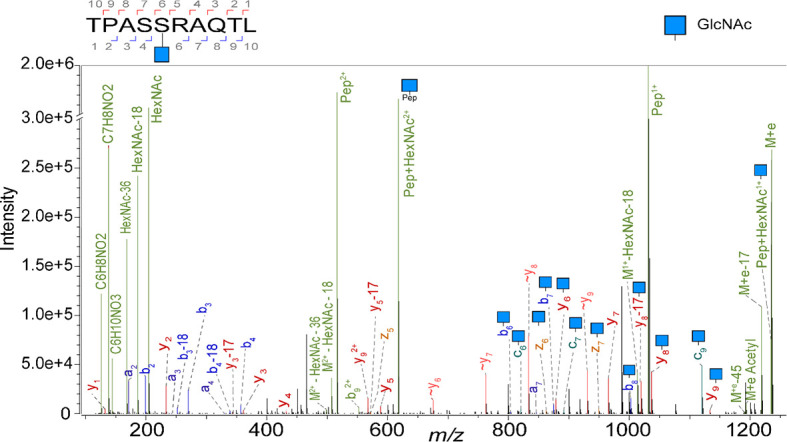
Illustrative EThcD mass spectrum of the peptide TPASgSRAQTL, originating from Spectrin, in its *O*-GlcNAcylated form, modified at P5. Highlighted in green are the oxonium ions. Fragment ions that elucidate the amino acid sequence and the glycosylation site are labelled as b, c, y and z. The fragment ions without modification are indicated with a wave dash.

Here, we first revisited the ligandome of the JY B-lymphoblastoid cell line, a model cell line used by several groups active in immunopeptidomics including ours (27, 33–35), as it has relative high expression of HLA complexes, and is homozygote in its HLA class I alleles, harbouring just HLA-A*02:01, HLA-B*07:02, and HLA-C*07:02 alleles. We have previously shown that several thousand of HLA peptides can be identified when analyzing JY cells. Here we attempted to characterize the glycosylated subset and report the detection of 23 unique *O*-GlcNAcylated HLA class I peptides. In our calculation we count each unique peptide just once, even when it is identified with multiple different PTMs. To boost the identifications we examined the occurrence of diagnostic oxonium ions (*m/z* 204, 186, 168, 144, 138) in the MS2 spectra, and used these features to trigger targeted analysis by ETD and stepped HCD, to boost the generation of fragment ions for identification of the glycopeptide backbone (17, 26). As described earlier (36), the relative abundance of the oxonium fragment ions was used to confirm *O*-GlcNAc assignment of all 23 *O*-GlcNAc HLA peptides identified, as opposed to being *O*-GalNAcylated. A representative EThcD spectrum from the *O*-GlcNAcylated TPASgSRAQTL peptide is shown in **Figure 1**, exposing both backbone fragments as well as glycan ions.

### Distribution of O-GlcNAcylated HLA Class I Peptides

**Figure 2:**
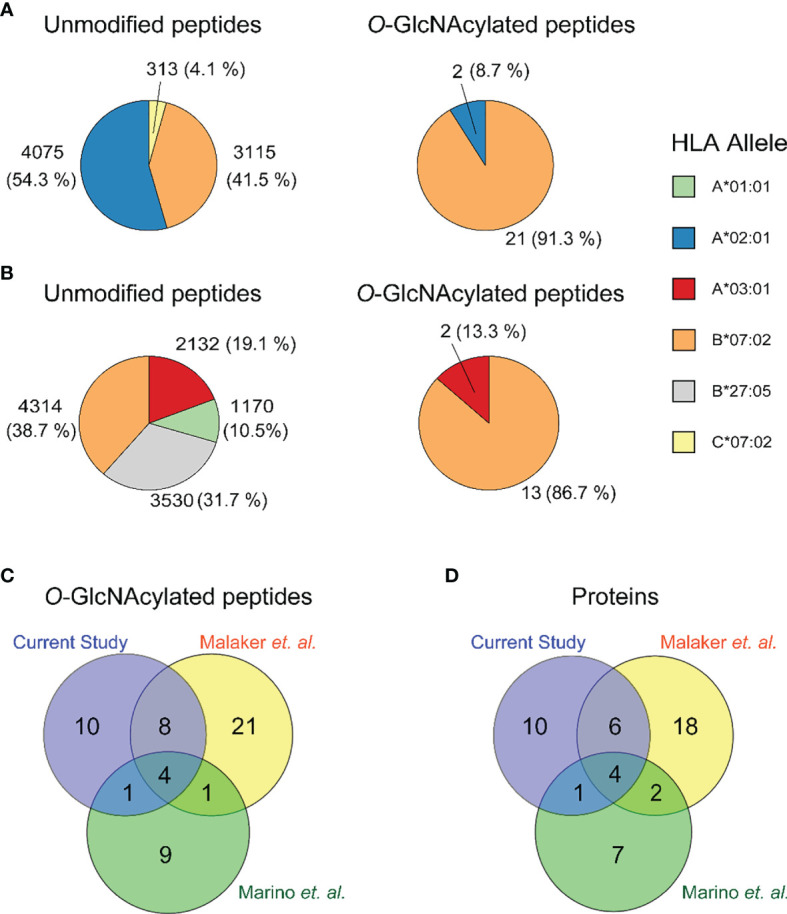
Distribution of the identified unmodified (left) and *O*-GlcNAcylated (right) HLA class I peptides over **(A)** the JY HLA alleles in the current study, and **(B)** all HLA alleles in the GR-LCL cell line **(C)** Overlap in identified peptides in the current study (purple), Malaker et al. (yellow) and Marino et al. (green). **(D)** Overlap in identified proteins in the current study (purple), Malaker et al. (yellow) and Marino et al. (green).

In the present ligandome analysis of the JY cells, we detected around ~ 8800 unique HLA class I peptides, and thus these 23 *O*-GlcNAcylated HLA peptides constitute in numbers just about 0.26% of the total ligandome. By examining the binding affinity to HLA-A*02:01, HLA-B*07:02 and HLA-C*07:02, we observed that 21 out of the 23 (91%) were predicted to be strong binders of the HLA-B*07:02 allele (**Figure 2A** and **Table 1**). This prevalence for HLA-B*07:02 was in striking contrast to the distribution of the unmodified HLA antigens, where 41% of all HLA peptides were predicted to be strong binders to HLA-B*07:02 (**Figure 2A**). The *O*-GlcNAcylated peptides in our JY dataset originated from just 21 non-redundant source proteins. We found this low number of detected *O*-GlcNAcylated peptides and source proteins quite surprising, as a recent compilation, termed the human ‘*O*-GlcNAc-ome’ database (37), described that in the human proteome at least 7000 different *O*-GlcNAcylation sites on cumulatively 5000 proteins could be present. Evidently, not many of these lead to an abundant presentation of *O*-GlcNAcylated HLA class I peptides. Around three quarters of the source proteins were of nuclear origin. We also observed that quite a few of the source proteins have been functionally annotated as binding to either DNA or RNA. This observation, although based on a small number of *O*-GlcNAcylated peptides detected in our dataset, seems to agree with the suggestion that *O*-GlcNAc modifications are critical in the regulation of transcriptional events (38).

**Table 1:** Compilation of O-GlcNAcylated HLA class I peptides identified in the current study by Marino et al. and Malaker et al.

#	Sequence	Length	UniProt ID	Source protein	Subcellular localization	Predicted HLA allele	Predicted Affinity (IC50 nM)	Start-Stop	Described at.
1	RPLSKgTVRF	9	P62280	40S ribosomal protein S11	Nucleus, Cytosol	HLA-B*07:02	35	132-140	CS
2	IVQAgTRTSL	8	Q6VMQ6	Activating transcription factor 7-interacting protein 1	Nucleus	HLA-B*07:02	992	831–838	(26)
3	NPVgSLPSL	9	Q6VMQ6	Activating transcription factor 7-interacting protein 1	Nucleus	HLA-B*07:02	69	881-889	(25)
4	EPSSTVVSL	9	O75129	Astrotactin-2	Membrane	HLA-B*07:02	445	1076–1085	(26)
5	HPMgSTASQV	9	Q13492	Clathrin assembly lymphoid myeloid leukemia	Nucleus, Golgi apparatus	HLA-B*07:02	25	345–353	(26)
6	VPTgTSSSL	8	Q14004	Cyclin dependent kinase 13	Nucleus	HLA-B*07:02	168	1284–1291	(26)
7	VPASSTSTL	9	Q9NYV4	Cyclin-dependent kinase 12	Nucleus	HLA-B*07:02	8	576-584	CS, (26)
8	LPKPANTSAL	10	Q2KHR2	DNA-binding protein RFX7	Nucleus	HLA-B*07:02	7	336-345	CS, (25)
9	TPIgSQAQKL^a^	9	Q96L91	E1A-binding protein p400	Nucleus	HLA-B*07:02	363	3024–3032	(26)
10	VPRSSSMVL	9	Q9H992	E3 ubiquitin-protein ligase	Nucleus, Cytosol	HLA-B*07:02	3	123-131	CS
11	APPSTSAAAL	10	Q86TM6	E3 Ubiquitin-protein ligase synoviolin	Endoplasmic	HLA-B*07:02	26	405–414	(26)
12	PPSTSAAAL	9	Q86TM6	E3 Ubiquitin-protein ligase synoviolin	Endoplasmic reticulum	HLA-B*07:02	742	405–414	(26)
13	APTSASNVM	9	P28324	ETS domain-containing protein Elk-4	Nucleus	HLA-B*07:02	22	238-246	CS
14	VLTSNVQTI	9	P32519	ETS-related transcription factor Elf-1	Nucleus	HLA-A*02:01	376	507-515	CS
15	RPPSSSQQL	9	Q8WYB5	Histone acetyltransferase KAT6B	Nucleus	HLA-B*07:02	70	1758–1766	(26)
16	LPRGSSPSVL	10	Q9GZN2	Homeobox protein TGIF2	Nucleus	HLA-B*07:02	5	105-114	CS
17	APTgSAAAL	8	Q86Z02	Homeodomain-interacting protein kinase 1	Nucleus, Cytoplasm	HLA-B*07:02	90	1116–1123	CS, (26)
18	APVgSSKSSL^a^	9	Q86Z02	Homeodomain-interacting protein kinase 1	Nucleus, Cytoplasm	HLA-B*07:02	22	1157–1164	CS, (26)
19	VPVgSVGPSL	9	Q86Z02	Homeodomain-interacting protein kinase 1	Nucleus, Cytoplasm	HLA-B*07:02	7	850–858	CS, (25), (26)
20	APFgSCRTEL	9	P32942	Intercellular adhesion molecule 3	Membrane	HLA-B*07:02	5	186-194	(25)
21	IPVgSKPLSL	9	Q16621	Leucine zipper protein 1	Cytoplasm	HLA-B*07:02	22	104–112	(26)
22	LPRNSTMM	8	Q9NPI6	mRNA-decapping enzyme 1A	Nucleus	HLA-B*07:02	61	335–342	(26)
23	HPSSTASTAL	10	Q96T58	Msx2-interacting protein	Nucleus	HLA-B*07:02	15	3041–3050	(26)
24	RPVgTASITTM^a^	10	Q9ULH7	Myocardin-related transcription factor B	Nucleus	HLA-B*07:02	19	927–936	(26)
25	IPVgSSHNSL^a^	9	Q06413	Myocyte-specific enhancer factor 2C	Nucleus	HLA-B*07:02	10	147–155	CS, (25), (26)
26	VPVgSNQSSL	9	Q14814	Myocyte-specific enhancer factor 2D	Nucleus	HLA-B*07:02	25	146–154	(26)
27	LPTgSLPSSL	9	P46531	Neurogenic locus notch homolog protein 1	Cell membrane	HLA-B*07:02	20	2464–2472	(26)
28	VPVSGgTQGL	9	P23511	Nuclear transcription factor Y subunit alpha	Nucleus	HLA-B*07:02	56	93-101	CS, (26)
29	MPVRPTgTNTF	10	Q7Z3K3	Pogo transposable element with ZNF domain	Nucleus, Cytoplasm	HLA-B*07:02	13	218–227	(26)
30	HPSSTAAVL	9	Q86XN7	Proline and serine-rich protein 1	–	HLA-B*07:02	13	740–748	CS, (26)
31	APRgTNGVAM	9	Q92567	Protein FAM168A	–	HLA-B*07:02	3	187–195	(26)
32	IPAVgTRSTI	9	Q96RT1	Protein LAP2	Nucleus, Plasma membrane	HLA-B*07:02	8	1066-1074	(25)
33	IPIgSLHTSL^a^	9	Q5JSZ5	Protein PRRC2B	–	HLA-B*07:02	11	1959–1967	(26)
34	APVgSASASV	9	Q9Y520	Protein PRRC2C	–	HLA-B*07:02	20	1807–1815	CS, (26)
35	MPSSSHGSM	9	Q15532	Protein SSXT	Nucleus	HLA-B*07:02	5	152-160	CS
36	IPTgSSVLSL	9	O15027	Protein transport protein Sec 16A	Endoplasmic reticulum	HLA-B*07:02	31	710–718	(26)
37	RPPQgSSSVSL	10	O15027	Protein transport protein Sec 16A	Nucleus	HLA-B*07:02	9	937–946	(26)
38	gSPRVTQTIAL	10	Q9BZA7	Protocadherin-11 X-linked	Plasma membrane	HLA-B*07:02	3	1186-1194	CS
39	RPPVTKASSF	10	Q9Y2K5	R3H domain-containing protein 2	Nucleus	HLA-B*07:02	26	341-350	CS, (25), (26)
40	RgSPTKSSL	8	Q96PK6	RNA-binding protein 14	Nucleus, Cytoplasm	–	–	243-250	CS
41	IPRPPIgTQSSL	9	Q9P2N5	RNA-binding protein 27	Nucleus, Cytoplasm	HLA-B*07:02	20	382–390	(26)^†^
42	RPPIgTQSSL^a,b^	11	Q9P2N5	RNA-binding protein 27	Nucleus, Cytoplasm	HLA-B*07:02	11	380-390	(25)^†^
43	RPgTPRGITL	9	Q7Z614	Sorting nexin-20	Nucleus, Cytoplasm	HLA-B*07:02	4	298-306	(25)
44	TPASSRAQTL	10	Q01082	Spectrin beta chain, non-erythrocytic 1	Cytoskeleton, Plasma membrane	HLA-B*07:02	9	2320–2329	CS, (25), (26)
45	APVgSPSSQKL	10	Q9NYB0	Telomeric repeat-binding factor 2-Interacting protein 1	Nucleus, Cytoplasm	HLA-B*07:02	70	200-209	(25)
46	KPPVgSFFSL	9	Q6PKC3	Thioredoxin domain containing protein 11	Endoplasmic reticulum	HLA-B*07:02	91	95–103	(26)
47	IPTgSARSML	9	Q8WXI9	Transcriptional repressor p66-beta	Nucleus	HLA-B*07:02	9	522-530	(25)
48	TPARSRgSKE	9	P62995	Transformer-2 protein homolog beta	Nucleus	HLA-B*07:02	2172	32-40	CS
49	VPEVgTKPSL	9	Q9UPQ9	Trinucleotide repeat-containing gene 6B protein	P-bodies	HLA-B*07:02	65	39-47	(25)
50	TPASSSSAL	9	Q9NPG3	Ubinuclein-1	Nucleus	HLA-B*07:02	5	875-883	CS, (26)
51	KPPTSQSSVL	10	Q5T6F2	Ubiquitin-associated protein 2	Nucleus, Cytoplasm	HLA-B*07:02	32	411–420	CS, (26)
52	VPVgSSASEL	9	Q7Z2W4-3	Zinc finger CCCH-type, antiviral 1	Nucleus, Cytoplasm	HLA-B*07:02	36	596–603	(26)
53	SVVTTVWGV	9	Q8NF64	Zinc finger MIZ domain-containing protein 2	Nucleus	HLA-A*02:01	12	40-48	CS
54	RVKTPTgSQSY	10	Q9Y2X9	Zinc finger protein 281	Nucleus	HLA-A*03:01	631	885-894	(25)^†^
55	RVKTPTgSQSYR	11	Q9Y2X9	Zinc finger protein 281	Nucleus	HLA-A*03:01	439	885-895	(25)^†^

The O-GlcNAcylated Thr or Ser is tagged with a “g” when unambiguously assigned by tandem MS data. ^†^Each of these two pairs of overlapping peptides were considered as a unique sequence. Peptides described in the current study are assigned as CS. ^a^These peptides were tested by Malaker et al. for immunogenicity. ^b^Methylated and O-GlcNAcylated peptidoform of this peptide was also tested for immunogenicity by Malaker et al.

Earlier, Marino et al. and Malaker et al. have reported on *O*-GlcNAcylated HLA class I peptides. We therefore consolidated our identifications, with their earlier data, to compile a comprehensive compendium of experimentally detected *O*-GlcNAcylated HLA class I peptides. Marino et al. were the first to report on a variety of glycosylated HLA class I peptides, immunoprecipitated and enriched using the pan-HLA-I**-**specific antibody (W6/32), from the GR B-lymphoblastoid cell line (25). This GR-LCL cell line is heterozygote in HLA class I alleles and harbors the HLA-A*01:01, A*03:01, B*07:02, B*27:05, C*02:02, C*07:02 alleles. In total Marino et al. detected 15 *O*-GlcNAcylated peptides with unique backbone sequences, in a full dataset of ∼15,000 HLA class I peptides (17, 25). Therefore, also in their data *O*-GlcNAcylated peptides constituted just around 0.1% of all HLA peptides. They observed that approximately 87% of the detected *O*-GlcNAcylated peptides were predicted to bind to HLA-B*07:02, whereas for the unmodified peptides this was just ~39% (**Figure 2B**). Another key feature of these *O*-GlcNAc HLA peptides was that most of them were derived from nuclear source proteins. All these findings are very much in line with our observations, although here we used the JY cell line.

Malaker et al. analyzed the HLA class I immunopeptidomes from blood cells of primary leukemia (chronic myeloid leukemia, acute myeloid leukemia and acute lymphoblastic leukemia) patients, relying on a HLA-B*07-specific antibody (ME1) to pull down HLA Class I complexes (26). Healthy T and B cells were isolated from normal spleen and tonsil biopsies from healthy donors. Additionally, they also included the JY cell line in their analysis, as a reference system. To optimize the detection of *O*-GlcNAcylated peptides they combined several different approaches, including HCD-MS/MS analysis of the HLA ligandome, making use of glycan specific neutral losses, an HCD triggered ETD approach like Marino et al., and a selective enrichment of esterified glycopeptides using an amino phenylboronic acid-derivatized affinity matrix to capture the glycopeptides. As in the other mentioned studies and ours, the relative glycan fragment ion intensities were used to confirm that all peptides identified were indeed *O*-GlcNAcylated. Notwithstanding these thorough and admirable efforts, and considering the multiple tissues, biopsies and cell lines investigated, Malaker et al. could identify and characterize only 34 unique glycosylated HLA class I peptides in total. Interestingly, most of the *O*-GlcNAcylated peptides (33/34) were found by them exclusively in the leukemia samples, and they reported solely one *O*-GlcNAcylated HLA peptide detected in the JY cells (as opposed to the 23 we report here). This increase in identifications of *O*-GlcNAcylated HLA Class-I peptides, when compared to the study of Malaker et al., could be due to multiple issues, such as the use of a more sensitive mass spectrometer, refined bioinformatics tools and a combination of hybrid fragmentation triggering methods. Moreover, Malaker et al. studied the JY cells just as a model system, focusing more on the primary cancer cells and tissues. As in the work of Marino et al., several (10 out of 34) *O*-GlcNAc peptides were also detected to carry one or more monosaccharide extensions (Gal and Gal-NeuAc).

Given the small number of *O*-GlcNAcylated HLA class I peptides detected in each of these studies, as well as in the current study, we had expected *a priori* that such small number of identifications might render the overlap in between these studies to be close to zero. Nonetheless, this was not the case, and instead, there was a substantial overlap in detected *O*-GlcNAcylated HLA class I peptides (**Figure 2C**) and their source proteins (**Figure 2D**). This was even more surprising, given that the source material in these three studies was completely different. Cumulatively, from the datasets described above we compiled a list of 55 unique O-GlcNAcylated HLA class I peptides (**Table 1**).

The potential role of O-GlcNAcylated peptides as neoantigens was investigated in depth by Malaker et al. Using a subset of both unmodified as well as *O*-GlcNAcylated HLA Class I peptides they assessed seven of the *O*-GlcNAcylated peptides detected in the leukemia cells. Five out of these seven HLA-B*07:02 glycopeptides were immunogenic. All healthy donors had immunity to at least one of the glycopeptides with strong responses similar to chronic viral antigens. They further assessed cytotoxicity responses in healthy donors towards a specific peptide harboring both methylation and *O*-GlcNAcylation. These latter HLA peptides invoked a significant T-cell activation. In contrast, no T-cell response was observed for the unmodified HLA peptide counterpart. Notably, we identified two out of the seven *O*-GlcNAcylated HLA-I peptides that were tested for immunogenicity by Malaker et al. also in our study on non-cancerous JY cells. These two peptides are APVgSSKSSL and IPVgSSHNSL In summary, these findings suggest that the specific T-cell response towards *O*-GlcNAcylated HLA-B*07:02 peptides may potentially represent an autologous immunoprotective mechanism against leukemia.

One of the most striking features examining all identified *O*-GlcNAc HLA class I peptides is that approximately 90% (50/55) peptides are predicted to be strong binders to HLA-B*07:02 allele. This finding was expected for the dataset of Malaker et al. as they used an antibody specific for HLA-B*07, but in Marino et al. and in the current study a pan-HLA antibody was used that has no specific preference for the HLA-B*07 allele. Thus, the compiled data hint at an overall preference for *O*-GlcNAcylated HLA class I peptides to be presented by HLA-B*07:02, regardless of the immunopeptidome isolation strategy.

### Preferential Loading of O-GlcNAcylated HLA Class I Peptides on HLA-B*07:02

**Figure 3:**
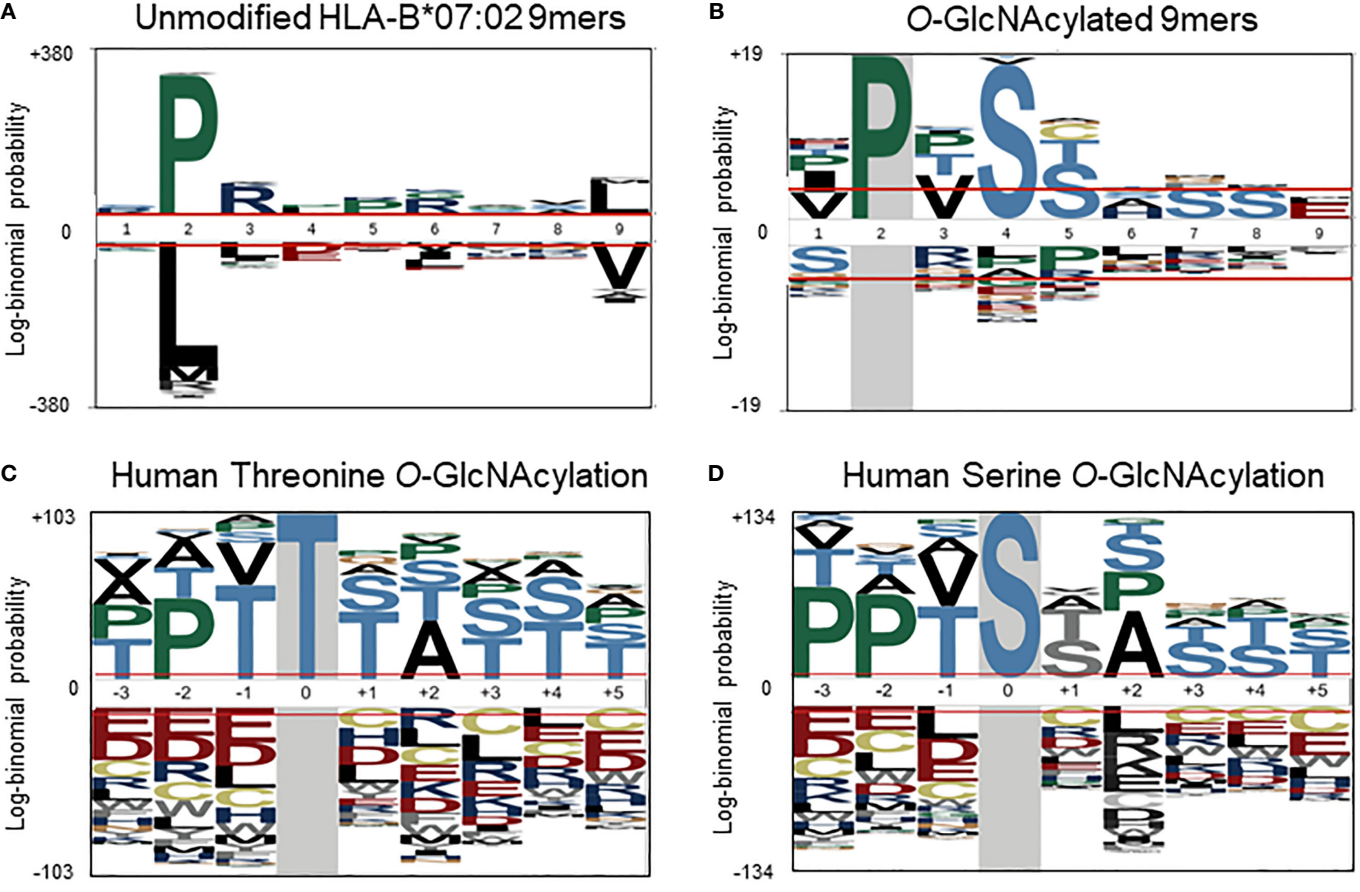
**(A)** Sequence motif (pLogo) of the in the current study detected unmodified HLA-B*07:02 peptides (9-mers) predicted as binders (% Rank < 2), with the clear anchoring positions Pro at P2 and Leu at P9. **(B)** Sequence motif of the *O*-GlcNAcylated HLA peptides (9-mers), with next to Pro at P2, Val at P3 and Ser at P4 and P5. Semi-consensus sequences based on the 7002 human O-GlcNAcylation sites for **(C)** Threonine and **(D)** Serine O-GlcNAcylation. (adapted from Wuff-Fuentes et al. (https://creativecommons.org/licenses/by/4.0/legalcode).

To further investigate a potential basis for the observed preferential presentation of *O*-GlcNAcylated peptides by the HLA-B*07:02 allele, we evaluated the peptide amino acid sequences of the 55 *O-*GlcNAcylated HLA peptides and compared them against the sequences of the unmodified peptides observed to bind the HLA-B*07:02 allele in the current dataset. Considering the site of modification, most *O*-GlcNAc sites were found in between position 3 to position 7 (P3-P7), with the highest incidence at position 4 (P4). Peptides presented by HLA-B*07:02 have a high preference for a proline residue at P2 position (**Figure 3A**). The peptide sequences of the 9-mer *O*-GlcNAcylated peptides (32 peptides in the compiled list) predominantly confirmed the presence of a proline residues at P2 along with preference for a valine residue at P3, a serine at P4 (72%, p-value < 0.05) and a serine or threonine residue at P5 (Ser 36%, p-value < 0.05) as illustrated in **Figure 3B**. Similarly sequences for the 10-mer *O*-GlcNAcylated peptides (12 peptides) were also rather consistent with a proline residue at P2 or P3, valine in P4 and serine/threonine residues at P4 to P6 (**Table 1**).

Although protein *O*-GlcNAcylation has been studied for decades, for a long time it was not clear that there could be a conserved substrate sequence motif. However, more recent structural (21, 39) and bioinformatic studies (37) have revealed a semi-consensus substrate motif for protein *O*-GlcNAcylation, namely P-P-(V/T)-g(S/T)-(S/T)-A (**Figures 3C, D**) (37). This motif matches well with the sequence motif compiled from our compendium of *O*-GlcNAcylated HLA class I peptides, where a dominant proline is observed (**Figures 3B, D**). It is quite conceivable that HLA-B*07 peptide antigens also naturally over-represent the *O*-GlcNAcylation motif, thereby resulting in preferential presentation of *O*-GlcNAcylated HLA class I peptides by the HLA-B*07 allele.

Compiling the combined dataset of *O*-GlcNAcylated HLA class I peptides from three recent studies, the substantial overlap in peptides detected in all or at least two of these studies was striking. Moreover, some other peculiar features were also repeatedly observed in all studies, for instance, the frequent co-occurrence of other PTMs, and the observation of glycan extensions next to the *O*-GlcNAcylation.

### Co-Occurring PTMs on O-GlcNAcylated Peptides (N-(di)Methylation, Phosphorylation)

**Figure 4:**
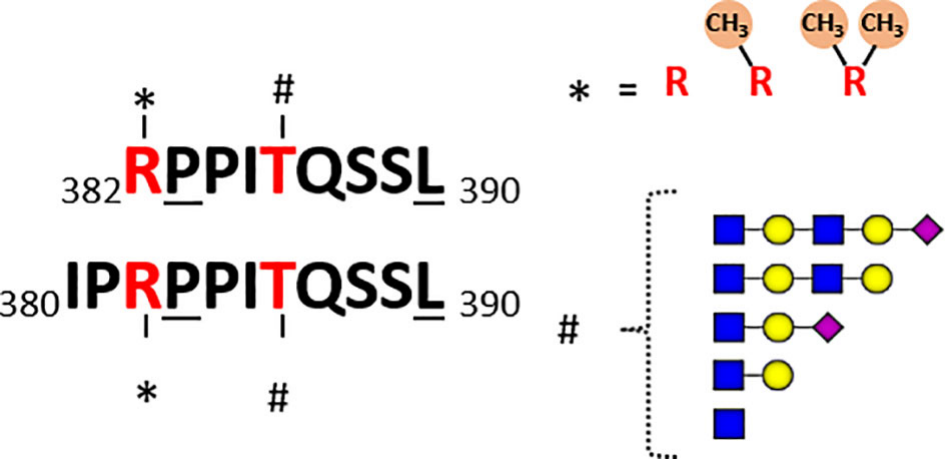
Schematic of detected co-occurring peptidoforms for the two overlapping unique HLA peptides detected, RPPITQSSL and IPRPPITQSSL, originating from RNA-binding protein 27. In the datasets of both Marino et al. and Malaker et al. several *O*-GlcNAcylated HLA peptides originating from the RNA binding protein 27 were reported. In Marino et al. the backbone sequence covered aa 380-390, whereas Malaker et al. observed the alike peptide covering aa 382-390. These peptides are likely HLA-B*07:02 binders, due to the underlined anchoring positions Pro at 2 and Leu at C-terminal position. In both datasets peptides were found with the Arg at 1 (3) being non-methylated, mono-methylated or di-methylated. The Thr at P5 (P7) was detected in several different glycoforms: non-O-GlcNAc, one GlcNAc, hexose-GlcNAc and even more extended glycans with mass shifts of 730 and 1021 Da.

Both Marino et al. and Malakar et al. observed glycan extensions on quite a few *O*-GlcNAcylated HLA peptides. For instance, the most immunogenic *O*-GlcNAcylated peptide identified by Malaker et al. was me-RPPIgTQSSL, originating from the RNA-binding protein 27 (aa 382–390), which contains both a methylated N-terminal arginine and an *O*-GlcNAcylated threonine at P5. Interestingly, killing of autologous B cells pulsed with methylated, O-GlcNAcylated, and the doubly modified HLA peptide was observed in their study, however not with the unmodified peptide. Potent T-cell response with the doubly modified peptide suggests that methylation may potentially further boost immunogenicity. This HLA peptide was detected in quite a few different peptidoforms in their studies **Figure 4**. Besides the GlcNAc (T5), mono-methyl (R1), they also observed the GlcNAc (T5), symmetric di-methyl (R1) and GlcNAc (T5), and asymmetric di-methyl (R1) + acetylGlcNAc (T5). Marino et al. also detected quite a few different *O*-GlcNAcylated peptides from the RNA-binding protein 27, albeit all within the N-terminal extended backbone IPRPPIgTQSSL (aa 380–390) **Figure 4**. They reported five different forms: non-*O*-GlcNAc, one GlcNAc (Thr5), hexose-GlcNAc and even more extended glycans with mass shifts of 730 and 1021 Da. Moreover, arginine (Arg3) was observed to be modified with a mono-methyl (Arg3) or asymmetric di-methyl (Arg3), both with and without the glycan modifications. Thus, although we define RPPgTQSSL and IPRPPIgTQSSL as one unique peptide in **Table 1**, these are presented as HLA ligands in a dozen of different “peptidoforms”. In general, not much is known about the RNA-binding protein 27, and the specific sequence motif presented as HLA peptides. Moreover, whether there is any cross-talk in between these PTMs is also not known.

Next, the peptides RVKpTPTgSQSY and RVKpTPTgSQSYR of the zinc-finger protein ZNF218 were also detected with both a phosphorylated Thr886 and an (extended) *O*-GlcNAc on Ser891. These peptides were also detected with mass extensions of 0, 203, 365, 656, 730, and 1021 Da, all indicative of a peptide backbone with extensions from 0 up to 6 glycan moieties. Both PTM sites have been reported previously, although no report is known if they co-occur in the source protein (40, 41). The proximity of *O*-GlcNAcylation and phosphorylation in RVKTPTSQSY may also hint at potential PTM crosstalk.

Finally, Malaker et al. reported glycosylation on the peptide IPVSSHNSL from the Myocyte-specific enhancer factor 2C protein, a transcription factor implicated in leukemogenesis (42), that was detected in 4 different glycoforms: GlcNAc (Ser4), double GlcNAc (Ser4, Ser5), single hexose-GlcNAc (Ser4), and an acetyl-GlcNAc (Ser4). This same peptide was also detected in Marino et al., although only in its *O*-GlcNAcylated form.

The origin of these additional glycan moieties is still puzzling. Most of the proteins observed to be decorated with these glycan extensions find their origin in the nucleus. Computational modelling of *O*-GlcNAcylated RPPVgTKASSF in complex with HLA molecule by Marino et al. revealed the glycan moiety was solvent exposed and not directly involved in the binding to the HLA Class I molecule groove. It is therefore plausible, as suggested earlier by Marino et al., that they may be first formed by proteasomal degradation as *O*-GlcNAcylated peptide, and then loaded onto an HLA class I molecule and extended by glycosyltransferases, starting with β1, 4 galactosyltransferase, in the Golgi, but this needs further validation. In general, the co-occurrence of so many peptidoforms of *O*-GlcNAcylated HLA Class I peptides raises the question whether all these forms have specific T-cell response and whether they are differentially immunogenic.

## Summary

Combining data from our new dataset of *O*-GlcNAcylated HLA peptides from JY cells, with two recent related studies, we were able to compile a concise list of *bone fide O*-GlcNAcylated HLA peptides, presented by a whole array of different cancer and non-cancerous cells. Our comprehensive analysis of the sequences of all these peptides revealed preferential presentation of O-GlcNAcylated HLA peptides by the HLA-B*07 allele. The proline at P2 position of this allele allows the semi-consensus sequence motif of *O*-GlcNAcylation to be presented prominently. This preferred rule of presentation by HLA-B*07 and HLA-B*07-like alleles may also be useful in predicting putative *O*-GlcNAcylated HLA peptide antigens. Moreover, although the number of *O*-GlcNAcylated HLA peptides detected are small in each of the three studies evaluated, the overlap between these diverse studies were substantially high. Several of the reported *O*-GlcNAcylated HLA peptides are presented in multiple peptidoforms, carrying either additional phosphorylation or arginine-(di)methylation, along with glycan extensions of between 0 to 6 more carbohydrate moieties. Whether such distinct peptidoforms have differential functionality in immune surveillance needs to be further addressed.

## Data Availability Statement

The datasets presented in this study can be found in online repositories. The names of the repository/repositories and accession number(s) can be found below: www.ebi.ac.uk/pride, PXD028874.

## Author Contributions

AH conceived the idea. AS-B performed the MS experiments. SM and AS-B analyzed the data and wrote the initial draft of the manuscript along with AH. SM and AS-B contributed equally to this manuscript. All the authors contributed to the article and approved on the submitted version.

## Conflict of Interest

The authors declare that the research was conducted in the absence of any commercial or financial relationships that could be construed as a potential conflict of interest.

## Publisher’s Note

All claims expressed in this article are solely those of the authors and do not necessarily represent those of their affiliated organizations, or those of the publisher, the editors and the reviewers. Any product that may be evaluated in this article, or claim that may be made by its manufacturer, is not guaranteed or endorsed by the publisher.
